# Combined Urinary Reconstruction During en Bloc Kidney Transplantation From a Pediatric Donor to an Adult Recipient: A Case Report

**DOI:** 10.7759/cureus.64489

**Published:** 2024-07-13

**Authors:** Pilar Leal-Leyte, Carlos U Camarillo-Sánchez, Daniel Zamora-Valdés

**Affiliations:** 1 Organ Transplantation, Naval Medical Center, Mexico City, MEX; 2 Hepatobiliary Sciences and Liver Transplantation, King Abdullah Specialized Children's Hospital, King Abdulaziz Medical City, Ministry of the National Guard Health Affairs, Riyadh, SAU

**Keywords:** deceased donor, case report, urinary reconstruction, en-bloc kidneys, kidney transplantation

## Abstract

Urinary reconstruction during en bloc kidney transplantation is challenging, with different techniques described. Here, we report a case of combined urinary reconstruction using modified Lich ureteroneocystostomy and ureteroureterostomy.

## Introduction

En bloc kidney transplantation increases the deceased donor kidney pool. Urinary reconstruction after en bloc kidney transplantation has been described using various techniques. Here, we report the successful management of vascular kinking after implantation of pediatric en bloc kidneys in an adult recipient through combined urinary reconstruction.

## Case presentation

**Figure 1:**
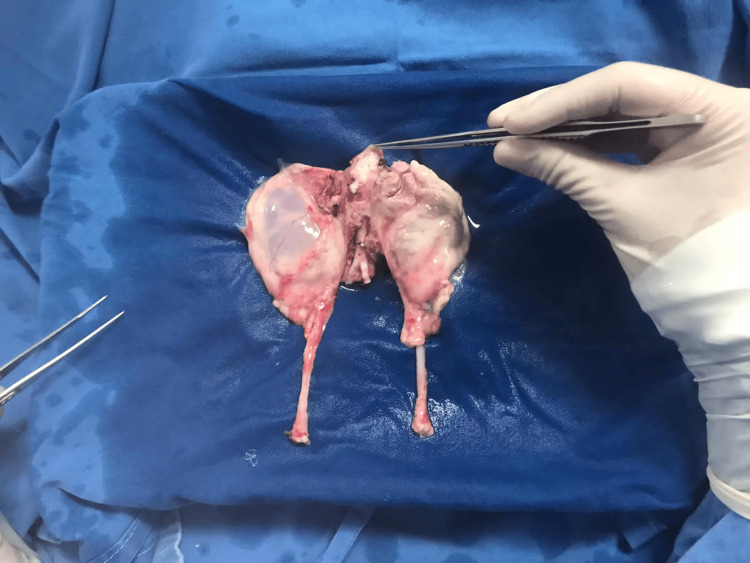
En bloc kidney allograft in the back table before preparation

The kidneys of an eight-month-old brain-dead donor (creatinine 0.1 mg/dL, KDPI 60%) were allocated to a 27-year-old male patient with idiopathic end-stage renal disease (EPTS, 2%). The donor weighed 10 kg; the right kidney was 4 cm, and the left 3 cm (Figure 1).

**Figure 2:**
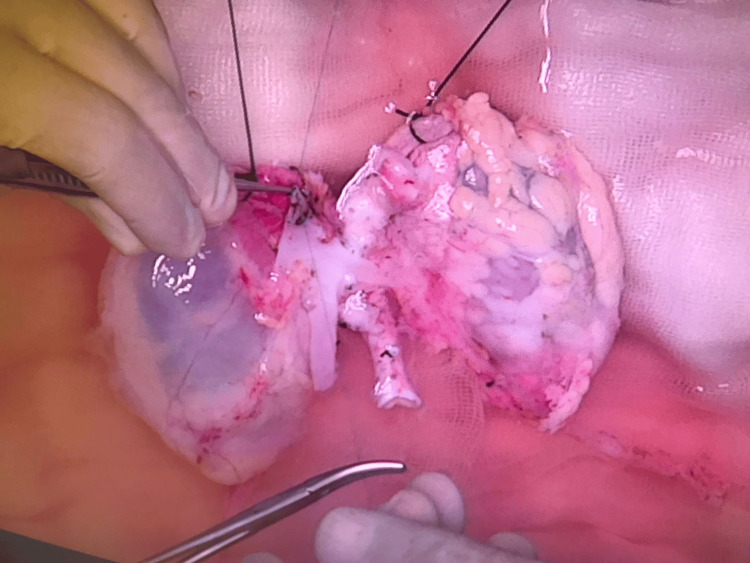
Caval affluents and aortic branches ligation and division, closure of the suprarenal inferior vena cava. Note that a 2-0 silk tie is sutured to the adrenal gland to be used as a handle during implantation

On the back table, the suprarenal aorta and inferior vena cava were sutured closed, small aortic branches and caval affluents were ligated, a Carrel patch using the common iliac arteries was created, and cavoplasty was performed using the common iliac veins (Figure 2).

**Figure 3:**
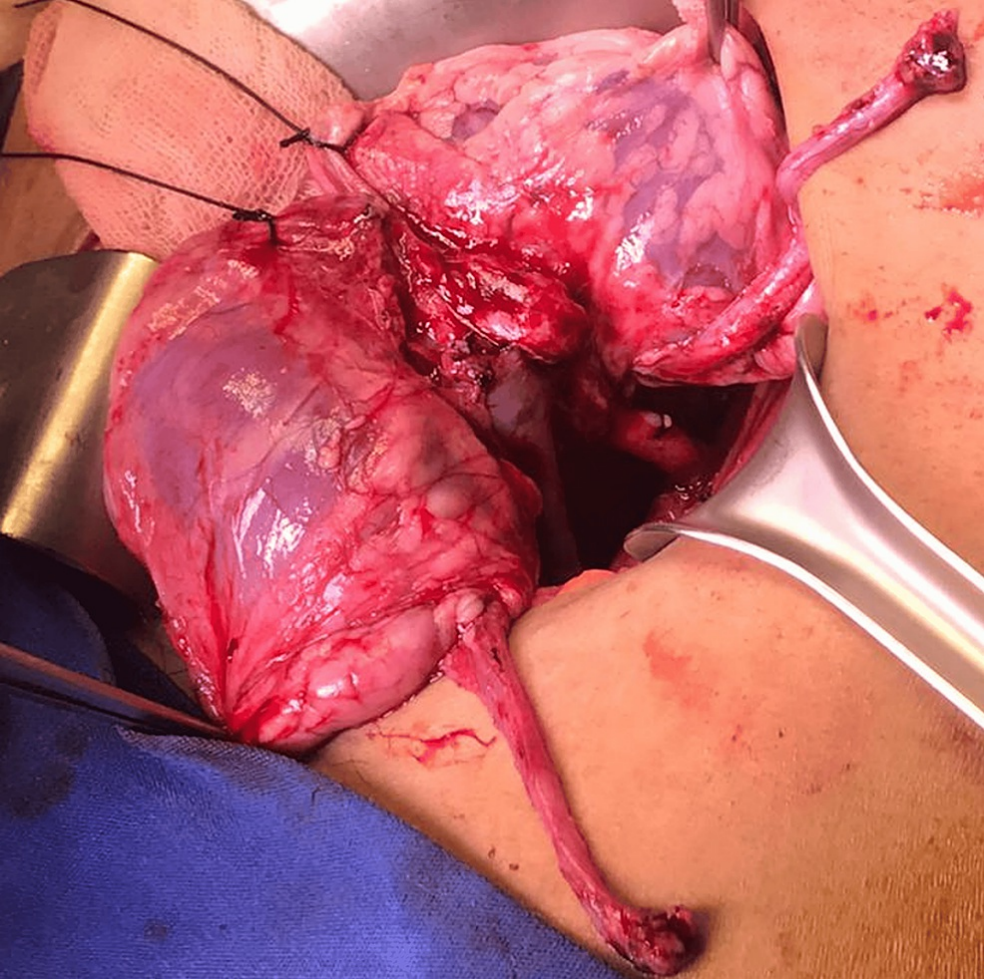
En bloc kidney allograft appearance 15 minutes after reperfusion

Two renal arteries were identified in the left kidney and three in the right kidney. The ureters were harvested and separated. The left external iliac vessels were exposed and mobilized using an extraperitoneal approach. The right kidney was caudal, and the left was cephalad. Distal cavoplasty and a Carrel aortic patch were implanted end-to-side into the external iliac vessels. The cold ischemia time was 13h, and the warm ischemia time was 32 min. Reperfusion was normal (Figure 3), and diuresis was observed.

Both ureters were pulled towards the bladder, but the vena cava kinked when the left allograft ureter was in contact with the bladder. At this point, we dissected and ligated the left native ureter, creating an end-to-end left ureteroureterostomy and a right modified Lich-Gregoir ureteroneocystostomy, both over 3.7 Fr ureteric stents. We tacked the en bloc allograft to the pelvic wall by using the adrenal glands and Gerota's fascia.

**Figure 4:**
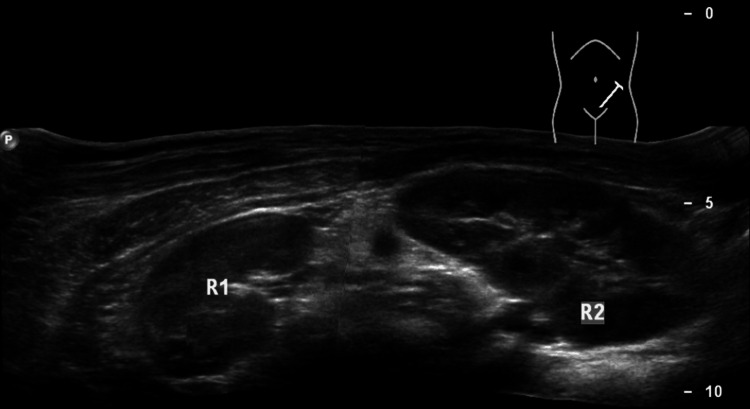
En bloc kidney allograft on ultrasound three years after transplantation

At the time of discharge, the renal allograft function was satisfactory. The ureteric stents were removed on day 21. The patient has excellent renal allograft function three years after transplantation (creatinine 0.9 mg/dL). The follow-up ultrasound findings were normal (Figure 4).

## Discussion

Small pediatric donors are underutilized for adult transplantation due to a history of complications such as arterial thrombosis, renal artery stenosis, urinary leakages, and hydronephrosis [1]. However, the long-term graft survival of pediatric en bloc pediatric kidneys in adult recipients is similar to that of living donor kidney transplantation [2]. Kidneys from very small donors (<12 months old) are implanted en bloc into adults to reduce the risk of vascular complications and to increase the graft mass, but when the size of the donor and the recipient are compatible, even single kidneys from infant donors (<13 kg) show excellent long-term outcomes [3].

During en bloc kidney transplantation from very small donors, the infrarenal cava and aorta are anastomosed to the iliac vessels to avoid the risk of complications arising from handling very small vessels and anastomoses requiring microsurgical techniques. We prefer to implant the kidneys en bloc in the reverse position to allow the renal pelvis and ureter to be anterior to the hilar vessels. We also performed vascular anastomoses to the left iliac vessels to allow for cephalad aortic anastomosis and caudal venous anastomosis, avoiding venous compression by the aorta when the allografts are positioned cephalad. We preserved the adrenal glands in the allografts and used them as handlers during implantation (Figure 2), and part of Gerota's fascia was used as an anchor during graft fixation to the pelvic wall.

Urologic complications after transplantation of en bloc kidneys from small pediatric donors have been described in 9.8-11% of the cases, with strictures representing 55% of the total complications and 95% of all complications present in the first five months after transplantation [4,5]. Extravesical ureteroneocystostomy exhibits a smaller risk of urological complications in regular kidney transplantation and is considered the current standard of care [6], with a meta-analysis favoring the Lich-Gregoir procedure over the Ledbetter-Politano and Taguchi techniques [7]. However, even when en bloc kidney transplants from pediatric donors are increasingly performed in adults, ureteral reconstruction in these cases is challenging, and there is no standard of care. Different extravesical urinary reconstruction techniques during en-bloc kidney transplantation have been described, including double ureteroneocystostomy, single conjoined side-to-side ureteroneocystostomy, and bladder patch anastomosis [8]. The bladder patch anastomosis was first performed in experimental transplantation and applied in humans 40 years later [9]. It is believed to have three advantages: decreased reconstruction complexity, bladder augmentation, and a physiological antireflux mechanism [10]. The risk of bladder patch anastomosis is ischemic injury of the distal tissue, leading to stricture.

Urinary ligation of the native ureters during kidney transplantation has been shown to be safe [11,12]. We took advantage of this evidence to adapt a urinary anastomosis to the left native ureter, avoiding kinking of the small vessels of our allograft without any significant morbidity to our patient after three years of follow-up.

## Conclusions

This case study illustrates two important adaptations. First, organs were offered to our center during the COVID-19 pandemic, in which most transplant centers were inactive in our country. We were faced with an unusual but excellent offer for a young adult without potential living donors and decided to go through with it. Second, after reperfusion, the vena cava kinked when the kidneys were in position for separate ureteroneocystostomies, leading us to adapt again for a separate anastomosis, which has proven to be safe after three years of follow-up. In conclusion, the current case demonstrates that versatility can lead to increased access to transplantation and good outcomes.

## References

[1] Andersen OS, Jonasson O, Merkel FK (1974). En bloc transplantation of pediatric kidneys into adult patients. Arch Surg.

[2] Sureshkumar KK, Habbach A, Tang A, Chopra B (2018). Long-term outcomes of pediatric en bloc compared to living donor kidney transplantation: a single-center experience with 25 years follow-up. Transplantation.

[3] Hoyer DP, Dittmann S, Büscher A (2020). Kidney transplantation with allografts from infant donors - small organs, big value. Pediatr Transplant.

[4] Fananapazir G, Tse G, Di Geronimo R (2020). Urologic complications after transplantation of 225 en bloc kidneys from small pediatric donors ≤20 kg: Incidence, management, and impact on graft survival. Am J Transplant.

[5] Mitrou N, Aquil S, Dion M (2018). Transplantation of pediatric renal allografts from donors less than 10 kg. Am J Transplant.

[6] Slagt IK, Klop KW, Ijzermans JN, Terkivatan T (2012). Intravesical versus extravesical ureteroneocystostomy in kidney transplantation: a systematic review and meta-analysis. Transplantation.

[7] Alberts VP, Idu MM, Legemate DA, Laguna Pes MP, Minnee RC (2014). Ureterovesical anastomotic techniques for kidney transplantation: a systematic review and meta-analysis. Transpl Int.

[8] Hiramoto JS, Freise CE, Randall HR (2002). Successful long-term outcomes using pediatric en bloc kidneys for transplantation. Am J Transplant.

[9] Lee S (1967). An improved technique of renal transplantation in the rat. Surgery.

[10] Kato T, Selvaggi G, Burke G (2008). Partial bladder transplantation with en bloc kidney transplant - the first case report of a 'bladder patch technique' in a human. Am J Transplant.

[11] Gallentine ML, Wright FH, Jr Jr (2002). Ligation of the native ureter in renal transplantation. J Urol.

[12] Goh BK, Dean PG, Cosio FG, Gloor JM, Prieto M, Stegall MD (2011). Bilateral native ureteral ligation without nephrectomy in the management of kidney transplant recipients with native proteinuria. Am J Transplant.

